# Early Insights into Implementation of Universal Screening, Brief Intervention, and Referral to Treatment for Perinatal Substance Use

**DOI:** 10.1007/s10995-023-03842-x

**Published:** 2023-11-17

**Authors:** Sarah E. Reese, Annie Glover, Stephanie Fitch, Joe Salyer, Valerie Lofgren, Clayton “Tersh” McCracken III

**Affiliations:** 1 Rural Institute for Inclusive Communities, University of Montana, Corbin Hall, Room 52, Missoula, MT 59812 USA; 2 Center for Population Health Research, University of Montana, Skaggs Building, Room 173, Missoula, MT 59812 USA; 3 Billings Clinic, 801 North 29th Street, Billings, MT 59101 USA; 4 School of Social Work, University of Montana, Jeanette Rankin Hall 026, 32 Campus Dr, Missoula, MT 59812 USA

**Keywords:** Universal screening, Perinatal, Substance use, Implementation, SBIRT

## Abstract

**Objectives:**

Perinatal substance use is a growing concern across the United States. Universal screening, brief intervention, and referral to treatment (SBIRT) is one systems-level approach to addressing perinatal substance use. The objective of this study is to assess early efforts to implement SBIRT in an outpatient obstetric clinic.

**Methods:**

The research team implemented universal screening with the 5 P’s screening tool. Providers then engaged patients in a brief intervention and referred to a care manager who then worked with patients via tele-health to connect patients with needed services. Feasibility was measured through the collection of aggregate data describing frequency of universal screening and referral to treatment. The implementation team met bi-weekly to reflect on implementation barriers and facilitators.

**Results:**

In the first year of implementation, 48.5% of patients receiving care in the clinic completed the 5 P’s screener at least once during the perinatal period. Screening occurred in a little over a quarter (26.5%) of eligible visits. Of the 463 patients that completed the 5 P’s at least once during the perinatal period, 195 (42%) unique patients screened positive (answered yes to at least one question).

**Conclusions for Practice:**

Early implementation efforts suggest this approach is feasible in this obstetric setting. Similar implementation studies should consider implementing universal screening for substance use and perinatal mood and anxiety disorders simultaneously; guide efforts using an implementation framework; invest resources in more intensive training and ongoing coaching for providers; and adopt strategies to track frequency and fidelity of brief intervention.

## Significance

Universal screening, brief intervention, and referral to treatment is a systems-level approach to addressing perinatal substance use, which is recommended by WHO, SAMHSA, USPSTF, and ACOG. Despite these recommendations, many clinics have not implemented SBIRT. There are few studies of implementation in settings that serve a high rate of rural and Indigenous patients. This study details the experiences and lessons learned from early efforts to implement SBIRT in an outpatient obstetric setting serving high rates of rural and Indigenous patients. This description is followed by a discussion of how findings will direct future implementation efforts in this and similar settings.

## Objectives

Perinatal substance use continues to be a concern across the United States (Rodriguez & Smith, 2019). Substance use during pregnancy can often be a sign of substance use disorder (SUD)^1^, a leading cause or contributing factor in pregnancy-associated deaths in Montana (Glover et al., 2020). Though pregnancy provides a unique opportunity to engage people in services for SUD (Le et al., 2019), fear of involvement with child protective services and social stigma related to substance use during pregnancy are barriers to pregnant people initiative prenatal care (Stone, 2015). People of color, in particular, face racism and discrimination in the healthcare and child welfare systems (Roberts & Nuru-Jeter, 2012), which may further deter engagement with healthcare systems. For example, American Indian/Alaskan Native (AI/AN) women are disproportionately impacted by the criminalization of perinatal substance use disorder (Simon et al., 2020). Additionally, women living in rural communities experience additional barriers to accessing care (Jumah, 2016), despite higher rates of substance exposure in utero compared to urban counterparts (Villapiano et al., 2017).

While some people cease using substances during pregnancy, many return to use in the postpartum period (Forray et al., 2015). For people who return to use, overdose is a risk for many due to a decreased tolerance to the substance (Kavanaugh, 2015; Smid et al., 2019). For many people with SUD, the postpartum period also presents unique risks for symptoms of postpartum mood and anxiety disorders (Corr et al., 2020), parenting stress (Rutherford & Mayes, 2019), and for many, especially during the COVID-19 pandemic, social isolation (Clark et al., 2021).

Universal screening is one effective approach to identifying pregnant people needing substance use-specific services and other services related to social determinants of health (Wright et al., 2016) that may improve outcomes and reduce healthcare costs (Courchesne-Krak et al., 2022). Research supports the effectiveness of universal screening, brief intervention, and referral to treatment (SBIRT) in primary care (Madras et al., 2009) and obstetric settings (Hostage et al., 2020; Ulrich et al., 2021). SBIRT is recommended by the World Health Organization (WHO, 2016), the Substance Abuse and Mental Health Services Administration (SAMHSA, 2021), the United States Preventive Services Task Force (Jonas et al., 2012), and the American College of Obstetricians and Gynecologists (ACOG, 2017). Self-report screening early and periodically during pregnancy can have clinical benefits for patients, for example, decreases in substance use and morphine treatment for newborns (Boden et al., 2021). A growing body of research suggests that SBIRT is feasible to implement in obstetric settings (Elertson & Schmitt, 2019; Hostage et al., 2020; Madras et al., 2009; Ulrich et al., 2021).

When implemented to fidelity, SBIRT reduces stigma and racial bias in screening, identifies people in need of care, and connects them with services. Implementation of universal screening remains challenging, even for states where it is mandated (Patel et al., 2021). Analysis of data from the Montana Pregnancy Risk Assessment Monitoring System (PRAMS) indicate potential racial bias in screening for perinatal substance use, with 91.7% of AI/AN women reporting they were asked during prenatal care about substance use, compared to 82.5% of White women in 2019 (Glover et al., 2020). The clinic where this implementation study occurred has a large rural service area with two Federal Indian Reservations.

The Empaths program in Billings, Montana is a pilot study designed to assess facilitators and barriers to implementing SBIRT in a rural outpatient obstetric clinic. The clinic serves high-risk patients who predominantly live in designated healthcare professional shortage areas, and the rate of families living below the poverty line in the service area (i.e., the county where this clinic is located and surrounding counties) ranges between 4.4% and 21.9% (Health Resources and Services Administration, 2023). This paper will report the preliminary findings on the implementation of SBIRT in an outpatient obstetric setting.

## Methods

The University of Montana Institutional Review Board approved this study protocol. The research team included a physician champion (Miech et al., 2018; Shaw et al., 2012), clinic leadership, the Empaths principal investigator (a Licensed Clinical Social Worker), and the principal investigator of the Montana Maternal Health Innovation Grant funded by HRSA, where Empaths is nested. The team surveyed existing obstetric clinic staff and identified a lack of services and expertise related to perinatal substance use in the clinic and broader health system. Providers reported they did not know what services and resources were available to patients using substances, did not feel confident in responding to a patient using substances, and did not know who to go to or how to refer patients to services. These perspectives match those reported by healthcare providers at large (Ordean et al., 2020). The research team then reviewed the existing literature and identified SBIRT as an approach to support providers and patients. At the end of the first year of the Empaths pilot, research questions and processes were developed in partnership with clinic leadership. Provider feedback informed the selection of a standardized screening tool and its application.

### Choosing a Validated Screening Tool

The American Congress on Obstetrics and Gynecology (ACOG, 2017) recommends universal screening for substance use be conducted at least once during the perinatal period and using a validated screening tool. ACOG suggests three tools: the 4-Ps (Chasnoff et al., 2007), the National Institute of Drug Abuse (NIDA) Quick Screen (Smith et al., 2010), and the CRAFFT (for women 26 years and younger; Chang et al., 2011). Ultimately, we chose to implement the 5Ps, an adaptation of Chasnoff’s 4Ps Plus perinatal substance use screening tool (Chasnoff et al., 2007; Ulrich et al., 2021), due to its high sensitivity, low cost, and preference from the clinic providers.

The 5Ps tool includes five questions: (1) *Did any of your parents have problems with alcohol or other drug use?* (2) *Does your partner have a problem with alcohol or drug use?* (3) *In the past, have you had difficulties in your life because of alcohol or other drugs, including prescription medication? (4) Before you were pregnant, did you have problems with alcohol or drug use?* and *(5) In the past month, did you drink beer, wine or liquor, or use other drugs?*

### Study Design

In the design of the Empaths pilot, we created a clear clinical pathway for referral by adding a care coordinator role to the clinic. Protocol for this study mandated all women receiving obstetric and postpartum care in the clinic complete the 5Ps (Chasnoff et al., 2007; Ondersma et al., 2019) at three time points: (1) their first obstetric visit, (2) a visit between 28 and 32 weeks, and (3) their first postpartum visit. Due to patients’ fear of self-reporting substance use, which is well-established in the literature (Stone, 2015), the team wanted to provide additional opportunities for patients to disclose after having time to build rapport with providers. Other clinics have implemented a similar timeline for screening (Ulrich et al., 2021).

A healthcare provider (e.g., obstetrician, midwife, registered nurse) then reviewed the results. If a patient answered yes to at least one question (indicating a positive screen), the provider engaged the patient in further conversation and referred them to the centralized care coordinator based on their assessment. The centralized care coordinator could then meet with the patient in person via a warm handoff or contact the patient at a later time via telephone. Given that this clinic serves women with barriers to accessing in-person care, the centralized care coordinator could work with the patient via telehealth to complete further assessment, conduct the brief intervention, and refer the patient to needed services (e.g., substance use, mental health, home visiting services).

As a part of the pilot, the clinic entered a contract with a statewide substance use and mental health provider that indicated a patient would be enrolled in at least one care option (i.e., medication management, peer support, outpatient mental health care, residential treatment) within 48 h of referral from the care coordinator. This team-based, system-level approach aimed to increase access to timely care and improve outcomes for pregnant and postpartum people.

Before the screening process began, clinic leadership and the physician champion provided in-person training to all clinic staff. Nurses and medical assistants were instructed to ask patients to complete a paper screener during the designated time points. All staff were given instructions to discuss any positive answers with the patient. The physician champion modeled how to initiate a conversation with a patient and how to introduce the care coordinator as a resource. All staff were instructed on where to place the completed screener, how to make a warm handoff if appropriate, and how to make a referral for care coordination.

Throughout the study, members of the implementation team engaged in specialized training to support this work. Members attended a Postpartum Support International advanced perinatal mental health training. Two implementation team members earned a Perinatal Mental Health Certification. Three implementation team members (including the care coordinator) trained to become recovery doulas—doulas who specialize in supporting families impacted by SUD. Three members also attended a Full Spectrum Indigenous Doula training, which focuses on the birthing experience and traditions of Indigenous communities. Clinic staff were invited to participate in Project ECHO (Extension for Community Healthcare Outcomes) clinics, which covered topics including perinatal substance use and cultural humility, Indigenous traditions regarding pregnancy and childbirth, healthcare policies impacting Indigenous communities, and trauma-informed care, among other topics. The Empaths care coordinator considered patients’ contexts when making referrals to services. Though a partnership exists with the statewide substance use and mental health provider, patients were never required to access services with this provider. The care coordinator worked individually with clients to learn about their needs, values, and preferences before connecting them to resources.

### Data Collection & Analysis

The Empaths implementation team met bi-weekly to discuss implementation barriers and facilitators. Informal referrals to the care coordinator began in January 2021, while the 5Ps screener was in the process of being approved by the clinic. Implementation of universal screening for substance use with the 5Ps began in April 2021. Between April 2021 and May 2022, we evaluated SBIRT adoption through aggregate data analysis describing the frequency of universal screening and referral to treatment. Over this period, the principal investigator recorded insights and observations from bi-weekly implementation team meetings. In December 2021, we also conducted a semi-structured focus group (*n* = 6) and interviews (*n* = 5) with clinic staff. These staff included medical assistants, nurses, obstetricians, and clinic leadership. Notes from the bi-weekly team meetings and the focus group, and the transcription of the interviews were coded using deductive thematic analysis (Braun & Clarke, 2006) to identify facilitators and barriers to implementation. The coder then brought these themes to the implementation team for member checking (Braun & Clarke, 2006).

## Results

Between April 2021 and May 2022, 954 unique patients received care at the obstetric clinic. Four-hundred-sixty-three (48.5%) patients completed the 5Ps screener at least once during the perinatal period. During this time, 728 first obstetric visits were mandated to perform universal screening. A patient completed a screener in one-third of those visits (*n* = 217). Six-hundred-thirty-one unique patients attended at least one visit during the 28- to 32-week period. About 20% (*n* = 128) of women who attended an appointment between 28 and 32 weeks completed a screener. Of the 627 patients who attended at least one postpartum visit, 182 (29.0%) completed a screener. We collected demographic data from the electronic medical record (see Table 1) for those who were screened. Nearly a third of patients (*n* = 134) screened were Medicaid-insured. The majority of those screened were White (*n* = 406; 87.7%); forty-five (9.7%) were AI/AN; and the remaining 12 patients were of another race or unknown (0.03%). The clinic’s patient population during this study period had a higher proportion of AI/AN patients than the state’s general population; approximately 6% of Montanans identify as AI/AN (United States Census Bureau, 2022).

**Table 1:** Demographics of patients screened

Variable	*n*	%
Insurance Coverage		
Medicaid	134	28.9
Other	329	71.1
Race		
White	406	87.7
American Indian/Native American	45	9.7
Other & Unknown	12	0.03
Community*		
Not rural	396	85.5
Rural	67	14.5

*based on Rural-Urban Commuting Area Codes

**Fig. 1:**
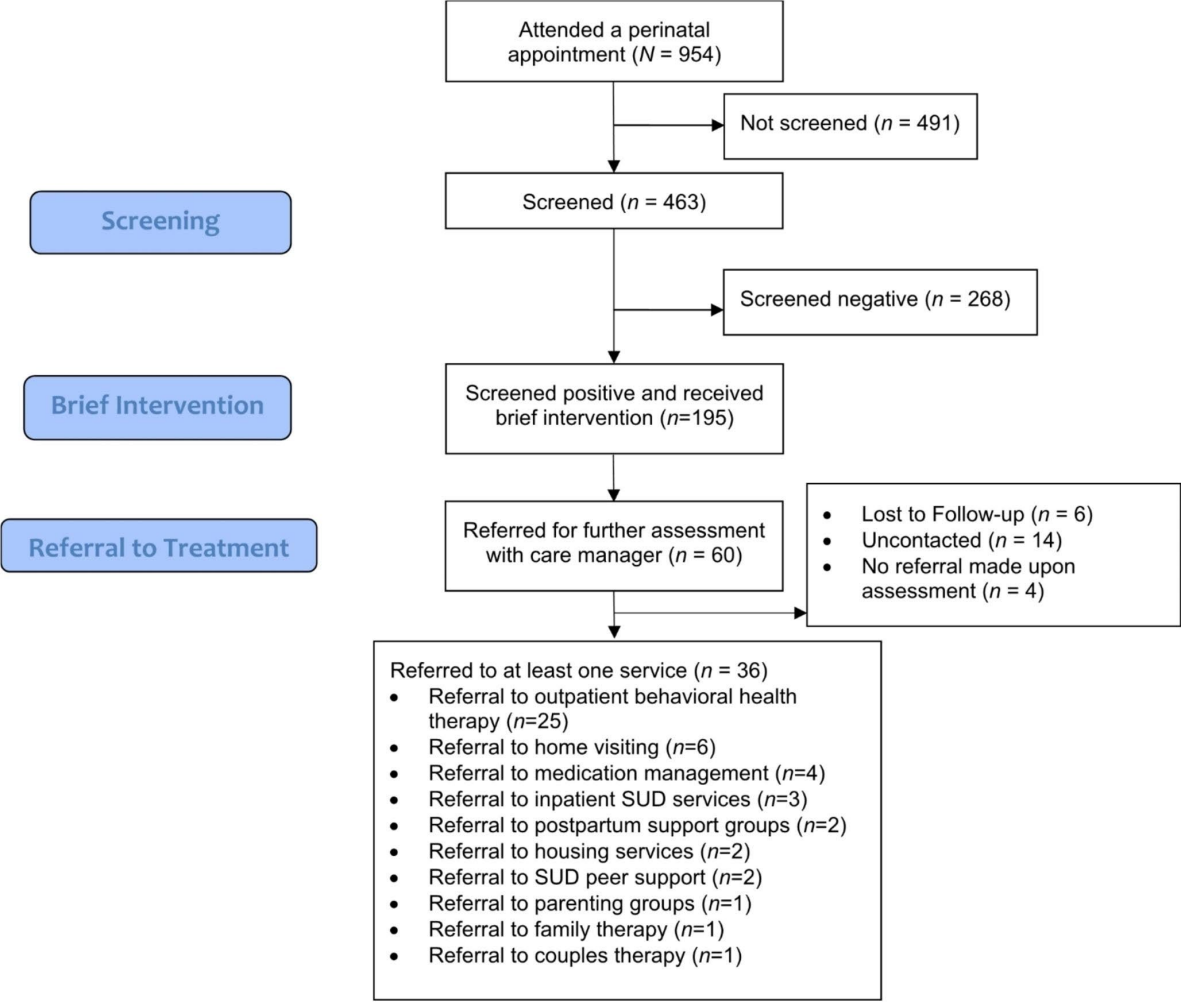
This flowchart demonstrates the number of patients who completed screening for substance use disorder using the 5Ps, received brief intervention, and were referred to treatment

Of the 463 patients who completed the 5Ps at least once during their pregnancy, 195 (42%) unique patients screened positive during pregnancy (answered yes to at least one question). Based on further assessment by the healthcare provider, 60 patients (30.8%) were referred to centralized care coordination. Six patients (10%) were unable to be contacted, and fourteen (23%) were not contacted. Forty patients were connected with care coordination, and 36 patients were referred to at least one service upon assessment. Among patients referred to at least one service, 67% actually received the service. Patients were referred to outpatient behavioral health therapy (*n* = 25), home visiting (*n* = 6), medication management (*n* = 4), inpatient SUD services (*n* = 3), postpartum support groups (*n* = 2), housing services (*n* = 2), SUD peer support (*n* = 2), parenting groups (*n* = 1), family therapy (*n* = 1), and couples therapy (*n* = 1). e.

### Barriers

The following factors were barriers to successful implementation of SBIRT: (1) lack of time and competing priorities during visits, (2) delays in integrating SBIRT into the electronic health record, (3) staff education and utilization, (4) challenges with coordination, (5) impact of SUD, and (6) the impact of the COVID-19 pandemic. Clinical staff reported they experienced a lack of time to complete all the tasks of obstetric and postpartum visits. Clinic staff described competing priorities during visits, and some reported they did not see screening for perinatal substance use as high of a priority as other tasks during visits. Staff also reported they often forgot to ask patients to complete a screener. Staff reported that adding a hard-stop alert (Powers et al., 2018) to the electronic medical record would be a helpful cue to ask patients to complete the screener.

There have been many barriers to incorporating SBIRT into the electronic health record, including adding the universal screening tool and documenting non-billed patient encounters with the care coordinator. The 5Ps screener has been added to the electronic medical record, but efforts are ongoing to add features such as a hard-stop alert and other design features to make the screener easier to navigate. Clinic leadership successfully advocated for adding notes from non-billable patient encounters to aid in care team communication.

There were initial efforts to educate and train clinic staff through an initial introduction during team meetings, followed by a “lunch and learn” training. As implementation began, the clinic staff expressed confusion and discomfort in asking patients to complete the screener and concerns about mandatory reporting of child abuse and neglect. To address these concerns, additional education and support were provided to clinic staff via an online training series and resources disseminated across a six-week period. Staff turnover impacted staff knowledge and implementation of SBIRT. New staff indicated they were unaware of the SBIRT process. This indicated a failure to fully onboard new clinic staff, provide ongoing education, training, and support, and efficiently integrate SBIRT into the workflow.

Challenges arose while coordinating patient care across organizational systems, for example, other agencies and child protective services. Strengthening relationships with key members of partnering organizations’ teams may help address care coordination challenges and facilitate communication between organizations. Symptoms of SUD itself - such as missing appointments - can present challenges in working with patients. (Miller & Ambrose, 2019). Fear and stigma can also prevent people from accessing prenatal care and engaging in treatment (Stone, 2015). Clinic staff reported engaging with patients and coordinating appointments can be challenging.

Challenges related to the COVID-19 pandemic influenced implementation efforts. The COVID-19 pandemic stretched the resources of this outpatient clinic and the larger healthcare system of which it is a part. However, these implementation efforts began approximately one year after the start of the pandemic, and it is possible that patients and healthcare providers alike were more open to telehealth options because of the increased comfort with technology due to the unique challenges the pandemic presented (Garfan et al., 2021). In addition to these barriers, facilitators were identified.

### Facilitators

Though universal screening was not achieved in the pilot program, the following factors were identified as facilitators of SBIRT implementation in this outpatient obstetric clinic: (1) engagement of a physician champion, (2) support of clinic leadership, (3) partnerships with other organizations, (4) staff expertise and community connection, and (5) technology. The physician champion brought an intimate understanding of the workings of the clinic and the challenges physicians and other medical providers encounter when providing day-to-day clinical care, such as limited time for patient encounters. The physician champion advocated for SBIRT with clinic leadership, physicians, and other medical providers and staff. He also provided insight when deciding which universal screening tool to implement and developing the study protocols. Since the implementation of perinatal substance use screening, he has provided informal support to providers and offered ongoing feedback as a part of the implementation team. Clinic leadership provided logistical support to integrate SBIRT into the existing visit protocols, provided direct feedback from the staff, and provided ongoing support to staff in the implementation process.

Though many settings struggle with low availability and accessibility of high-quality services to support pregnant women with SUD, the formal agreement between the clinic and a community substance use provider expedited patients’ access to needed services. This financial agreement with the community substance use provider guaranteed patients would connect with one level of care within 48 h (e.g., peer support, outpatient, or inpatient care). Currently, the clinic does not have this type of arrangement with other organizations but may consider creating these moving forward.

Finally, access to technology has been essential to implementation efforts. Given challenges related to COVID-19 precautions (e.g., protocols that limit in-person visits to reduce spread) and other barriers to meeting in person, such as transportation, telehealth technologies allowed the care coordinator to provide services virtually. Technology was used to schedule and conduct patient visits without the constraints of transportation, childcare, or other barriers.

## Conclusions for Practice

Overall, the Empaths pilot program did not achieve universal screening, and there were fewer than anticipated referrals to SUD treatment than expected during this implementation period. This clinic, like many healthcare settings, including obstetric settings in the United States, encountered challenges in implementing SBIRT (McNeely et al., 2018; Patel et al., 2021; Rahm et al., 2015), including necessary changes to workflow (Hunt et al., 2022), limited time and competing priorities during patient appointments (Cook, Green, de la Ronde, Dell, Graves, Morgan et al., 2017; Oni et al., 2020; Palmer et al., 2019; Van Hook et al., 2007), insufficient training and support for clinic staff (Anderson et al., 2010; Oni et al., 2020; Palmer et al., 2019; Van Hook et al., 2007), discomfort among clinic staff in implementing SBIRT for substance use in the perinatal period (Hand et al., 2019), and staff turnover (Vendetti et al., 2017). The screening rate for a visit between 28 and 32 weeks was lower than the rates at the first prenatal and postpartum visits, which may indicate less success integrating screening into the workflow for that time point, in particular. Almost a quarter of patients were not contacted for care coordination (*n* = 14), which may indicate issues in communication among the clinic team.

A much smaller number of women were referred for SUD services than anticipated, based on the prevalence of perinatal SUD. This may be related to the fear and distrust many women and pregnant people have related to substance use disclosure (Stone, 2015), stigma, and readiness for treatment at the time they were screened and assessed (Prochaska & Velicer, 1997). People who use substances during pregnancy may deny drug use when screened due to fear of negative consequences (Roberts & Nuru-Jeter, 2010; Stone, 2015; Woodruff et al., 2021). Some researchers argue that universal screening for substance use in pregnancy may deter engagement in prenatal care (Roberts & Nuru-Jeter, 2010). In future studies, we hope to better measure and describe patients’ experiences with screening as well as how the screening approach impacts patients’ willingness to disclose perinatal substance use. Additional training on trauma-informed care for staff may also create an environment where pregnant and postpartum people are more likely to disclose substance use (Sperlich et al., 2017).

Initially, we designed this study to meet providers’ needs and address racial bias in perinatal substance use screening, which was evident through state PRAMS data. We have yet to meet our universal screening goal and are unable to calculate the rates of screening between patients with different racial identities because we did not gather demographic data for those who did not complete a 5Ps screening tool. We can compare the racial identity of those who were screened (87.7% White, 9.7% AI/AN, 0.03% other or unknown) with demographic data from Montana gathered in April 2020. At that time, 88.9% of residents identified as White and 6.7% as AI/AN (United States Census Bureau, 2022).

To address barriers to implementation, improve screening rates, and overall implementation of SBIRT, we have initiated a more structured quality improvement approach guided by the Institute for Healthcare Improvement’s Model for Improvement (Langley et al., 2009). With specialized training and quality improvement coaching, the implementation team has engaged in process mapping and will initiate plan-do-study-act cycles to create a more sustainable SBIRT workflow for the clinic. We also plan to draw from the suite of resources created by ACOG for educating providers on perinatal mental health and a guide to integrating perinatal mental health care into obstetric practice. We are making efforts to engage all clinic staff more meaningfully to gain buy-in (Konkle-Parker et al., 2023). To address turnover, we plan to implement training protocols into the onboarding process to ensure all clinic staff feel prepared to implement SBIRT and provide ongoing SBIRT training and support based on staff feedback.

This evaluation of the Empaths pilot program adds to the limited research on the implementation of SBIRT in obstetric settings (Chasnoff et al., 2007; Cook, Green, de la Ronde, Dell, Graves, Ordean et al., 2017; McNeely et al., 2018; Ulrich et al., 2021). Strengths of the Empaths pilot project include the use of a screening tool validated for the perinatal period, including a practice champion on the implementation team, and developing relationships with referral partners.

The perinatal period is a unique opportunity to identify SUD and connect people to care (Le et al., 2019). SBIRT is an effective systems-level approach to addressing perinatal substance use and can reduce racial bias in screening practices, yet implementation of SBIRT presents challenges in healthcare settings, including obstetric settings (McNeely et al., 2018; Oni et al., 2020; Palmer et al., 2019; Van Hook et al., 2007; Vendetti et al., 2017). The inability to fully implement SBIRT in the Empaths Program may be related to the failure to set up a resilient system of care (i.e., a process for universal screening that is not impacted by changes such as staff turnover) and a lack of effective training and ongoing support for providers. Clinics implementing SBIRT might consider implementing a structured quality improvement process; providing comprehensive and ongoing education, training, and support to clinic staff; and integrating the SBIRT process into the electronic medical record in a meaningful way.

## Data Availability

N/A.

## Footnotes

1 Substance use disorder is defined by the fifth edition of the Diagnostic Statistical Manual (American Psychiatric Association, 2013) as the escalating use of a substance or multiple substances (e.g., alcohol, cannabis, methamphetamine, nicotine, opioids) despite the desire to stop using and continued use despite negative consequences to functioning at home, work, or school, and negative impacts on relationships, activities, safety, and physiological and psychological health. These symptoms are accompanied by tolerance, needing more and more of the substance to achieve the desired effect, as well as withdrawal, negative physical and psychological symptoms related to not using the substance(s) (American Psychiatric Association, 2013).

## Declarations

### Data Deposition

N/A.
